# Heat Dissipation for Microprocessor Using Multiwalled Carbon Nanotubes Based Liquid

**DOI:** 10.1155/2013/305957

**Published:** 2013-12-24

**Authors:** Bui Hung Thang, Pham Van Trinh, Nguyen Van Chuc, Phan Hong Khoi, Phan Ngoc Minh

**Affiliations:** ^1^Institute of Materials Science, Vietnam Academy of Science and Technology, A2 Building, 18 Hoang Quoc Viet Road, Cau Giay District, Hanoi 122102, Vietnam; ^2^Center for High Technology Development, Vietnam Academy of Science and Technology, 2B Building, 18 Hoang Quoc Viet Road, Cau Giay District, Hanoi 122102, Vietnam

## Abstract

Carbon nanotubes (CNTs) are one of the most valuable materials with high thermal conductivity (2000 W/m · K compared with thermal conductivity of Ag 419 W/m · K). This suggested an approach in applying the CNTs in thermal dissipation system for high power electronic devices, such as computer processor and high brightness light emitting diode (HB-LED). In this work, multiwalled carbon nanotubes (MWCNTs) based liquid was made by COOH functionalized MWCNTs dispersed in distilled water with concentration in the range between 0.2 and 1.2 gram/liter. MWCNT based liquid was used in liquid cooling system to enhance thermal dissipation for computer processor. By using distilled water in liquid cooling system, CPU's temperature decreases by about 10°C compared with using fan cooling system. By using MWCNT liquid with concentration of 1 gram/liter MWCNTs, the CPU's temperature decreases by 7°C compared with using distilled water in cooling system. Theoretically, we also showed that the presence of MWCNTs reduced thermal resistance and increased the thermal conductivity of liquid cooling system. The results have confirmed the advantages of the MWCNTs for thermal dissipation systems for the **μ**-processor and other high power electronic devices.

## 1. Introduction

Research on thermal dissipation materials for high power electronic devices has been receiving a special interest of the scientists and technologists. Besides finding new materials and technologies to increase component density and process speed of electronic and optoelectronic devices, it is very important to find new materials and appropriate configuration to accelerate the thermal dissipation [1].

In recent years, many approaches can improve the cooling system performance; the most feasible one is to enhance the heat transfer (dissipation) performance through the working fluid without modifying the mechanical designs or key components. Researchers have recently shown much interest on the issue of nanofluid thermal properties [2].

Nanofluid concept is employed to designate a fluid in which nanometer sized particles are suspended in conventional heat transfer base fluids to improve their thermal physical properties. Nanoparticles are made from various materials, such as metals (Cu, Ag, Au, Al, and Fe), oxide ceramics (Al_2_O_3_ and TiO_2_), nitride ceramics (AlN, SiN), carbide ceramics (SiC, TiC), semiconductors, and carbonnanotubes and composite materials such as alloyed nanoparticles or nanoparticle core polymer shell composites. It is well known that conventional heat transfer fluids, such as oil, water, and ethylene glycol, in general, have poor heat transfer properties compared to those of most solids. Nanofluids have enhanced thermophysical properties such as thermal conductivity, thermal diffusivity, viscosity, and convective heat transfer coefficients compared with those of base fluids like oil or water [3].

Carbon nanotubes (CNTs) are one of the most valuable materials with high thermal conductivity (2000 W/m · K compared to thermal conductivity of Ag 419 W/m · K) [4–6], On the other hand, MWCNTs could be dispersed in certain solutions via ultrasonication, surfactant treatment, or functionalization methods [7]. This suggested an approach in applying the MWCNTs in grease or liquid for thermal dissipation systems for computer processor and other high power electronic devices [8–16]. In this paper, we present the results of application of MWCNTs based liquid for a personal computer processor.

## 2. Experiment

MWCNTs were produced at Vietnam Academy of Science and Technology by thermal CVD technique. Fe(NO_3_)_3_ · 9H_2_O was used as precursor for the catalytic Fe nanoparticles. CaCO_3_ was mechanically mixed with this salt to get the catalyst. The concentration of Fe in the Fe(NO_3_)_3_ · 9H_2_O/CaCO_3_ mixture was 5 wt.%. Approximately 3 g of catalyst was uniformly dispersed in a stainless-steel boat and the boat was settled into the center part of a quartz reactor (with a diameter of 3.5 cm and a length of 120 cm) placed horizontally inside a tube furnace (20 cm longheating zone). Nitrogen flow of 300 sccm (standard cubic centimeters per minute) was supplied in the whole process. A hydrogen flow of 100 sccm was let into the reactor at 800°C. After 10 minutes for the hydrogen reducing reaction, the decomposition of a 50 sccm acetylene flow was carried out for 30 minutes. The reactor was then cooled down to room temperature in the N_2_ medium and the CVD product was removed. For the CNT purification, the CVD product was sonicated in dilute HCl acid (15%) for 1 h at room temperature, then filtered to remove the support, washed with distilled water, and dried at 130°C for 20 h [17]. The diameter and length of the grown MWCNTs used in this experiment were 15–90 nm and several ten *μ*m, respectively.

MWCNTs were functionalized with carboxyl (-COOH) by treatment in the mixture of hot acid (HNO_3_ : H_2_SO_4_, 1 : 3) at 60°C in 6 h and then dried in argon atmosphere at 80°C for 24 h [18, 19]. In order to disperse MWCNTs-COOH in liquid, we used the Tween-80 surfactant and Hielscher Ultrasonics Vibration instrument. The MWCNT-COOH was dispersed in liquid with concentration that was from 0.2 to 1.2 g/L [20].

**Figure 1:**
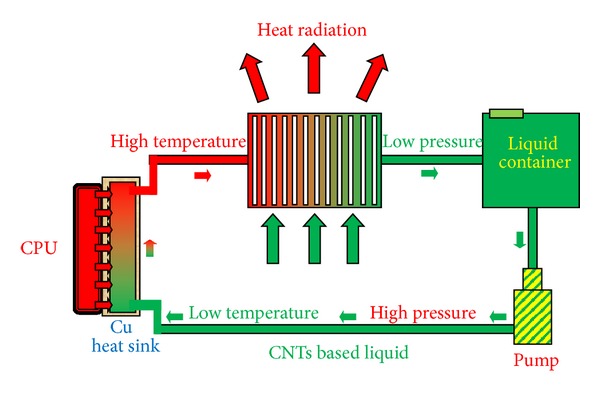
Scheme of cooling system for CPU using CNTs based nanoliquid.

Figure 1 is a schematic view of the thermal dissipation system for the computer processor using CNTs based liquid. In this system, the copper substrate was set to directly contact with processor and the tracks inside the copper substrate were fabricated to allow liquid flows through it and absorb heat from processor. The CNT based liquid was pumped into the Cu substrate with 2 cm^3^/s of flow rate. The volume of the liquid tank was 500 mL. The environmental temperature was kept at 15°C for all measurements. The thermal dissipation efficiency and thermal response of the system were evaluated by directly measuring the temperature of the *μ*-processor using dedicated software and a built-in temperature sensor inside the *μ*-processor [21]. We chose a personal computer with the following configuration: Intel Pentium IV, 3.066 GHz, 512 MRAM, 80 GB Hard-disk, and Window XP Service Pack 2 Operating System for the measurement [20]. The temperature of the *μ*-processor was measured by using Speed Fan 4.3.3 software and the *μ*-processor was pushed to operate in full load (100% usage of the processor) by using Stress Prime 2004 ORTHOS software [16].

## 3. Results and Discussions

MWCNTs were functionalized with carboxyl (COOH) functional groups. The existence of carboxyl functional groups was bonded to the ends and sidewalls. MWCNTs-COOH were demonstrated by Raman and FTIR spectra as reported in [19]. The Raman spectra were clearly seen that the two bands around 1593 and 1328 cm^−1^ in the spectra were assigned to the tangential mode (G-band) and the disorder mode (D-band), respectively. The D-band intensity was increased in the modified MWCNTs compared to raw MWCNTs. The peak intensity ratios (*I*_*D*_/*I*_*G*_ = 1.60) at D-band and G-band for the functionalized MWCNTs exceeded those of raw MWCNTs (*I*_*D*_/*I*_*G*_ = 1.27). This result indicates that some of the sp^2^ carbon atoms (C=C) were converted to sp^3^ carbon atoms (C–C) at the surface of the MWCNTs after the acid treatment in HNO_3_/H_2_SO_4_. The existence of carboxyl (COOH) functional groups bonded to the ends and sidewalls was demonstrated by FTIR spectra. Its shows some important peak after MWCNTs was treated by mixture of H_2_SO_4_ and HNO_3_. The vibration of O–H bonding in carboxyl group was shown on peak 3431.81 cm^−1^. It expanded more if compared with O–H bonding of H_2_O. Peak 1707.31 cm^−1^ showed the existence of vibration of C=O bonding in carboxyl group. This rendered important to prove the existence of carboxyl (COOH) functional groups appeared due to the oxidation resulting from nitric and sulfuric acids. It clearly shows that the kinds of acids functionalized the surface of MWCNTs [19].

**Figure 2:**
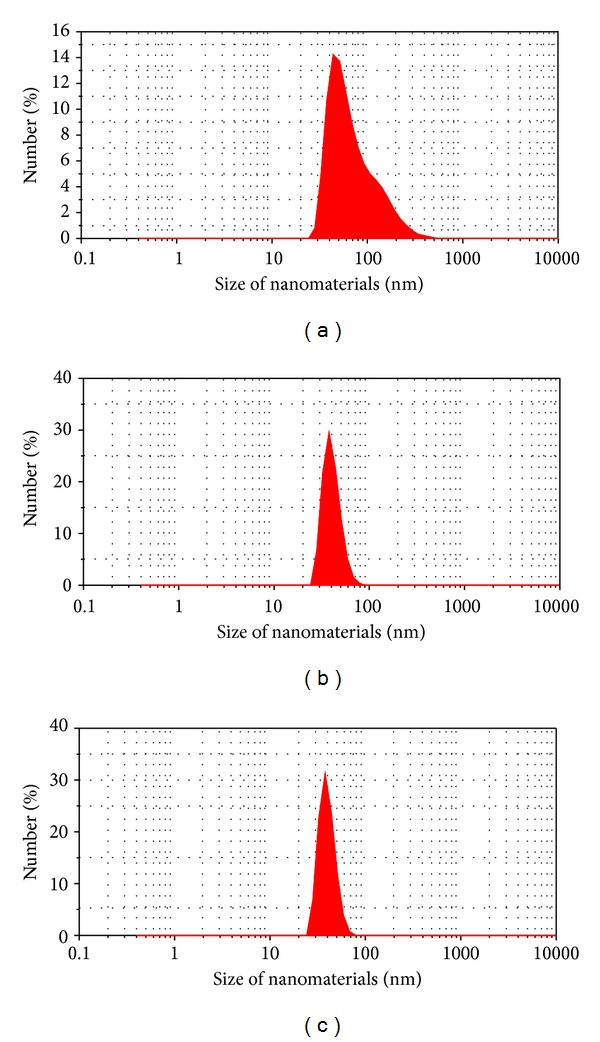
Spectra of the MWCNTs size distribution in water by number: (a) 20-minute ultrasonic vibration; (b) 30-minute ultrasonic vibration; and (c) 40-minute ultrasonic vibration.

**Figure 3:**
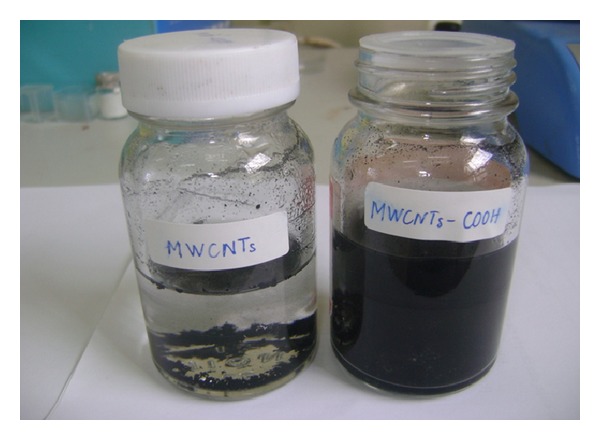
The typical image of MWCNTs-COOH was well dispersed in water after 30-minute ultrasonic vibration.

In order to evaluate the dispersion of MWCNTs in liquid, we used the Malvern Zetasizer Nano ZS Instrument.  Figure 2 shows spectra of the MWCNTs size distribution in distilled water by number.  Figure 2(a) showed that with 20 minutes of ultrasonic vibration time, the MWCNTs were still gathering into bundles with the size distribution from 20 nm to 450 nm. In the case of 30-minute or 40-minute ultrasonic vibration time, the MWCNTs-COOH well dispersed in water shown in Figures 2(b) and 2(c). The spectra of the MWCNTs size distribution by number in Figures 2(b) and 2(c) matched with 15–90 nm of the diameter of MWCNTs. The results showed that the ultrasonic vibration time required is more than 30 minutes for well-dispersion of MWCNT in water, so we chose 30 minutes of ultrasonic vibration time for subsequent experiments.  Figure 3 shows that the MWCNTs-COOH well dispersed in water after 30-minute ultrasonic vibration by using Tween-80 surfactant.

**Figure 4:**
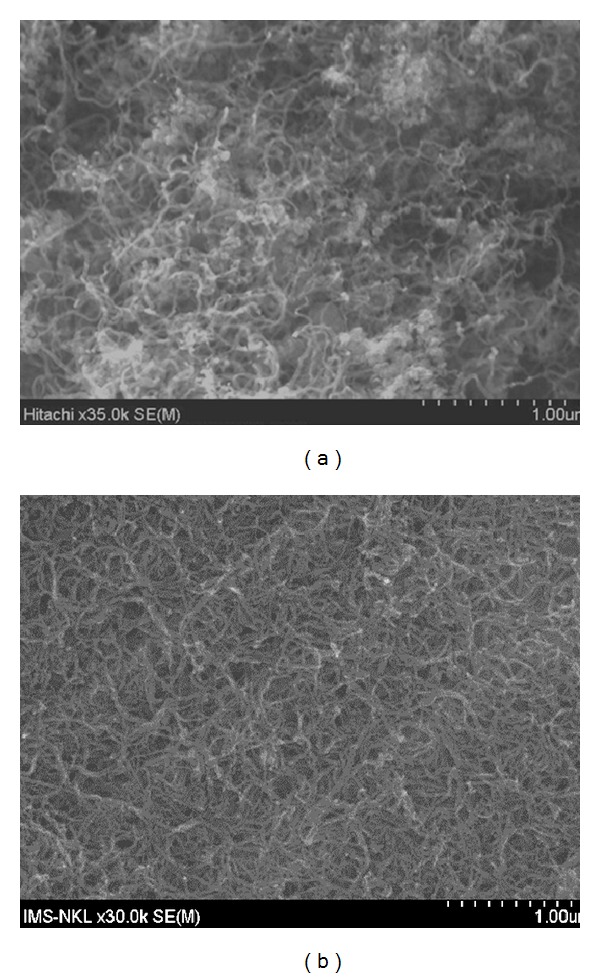
The typical SEM image of the MWCNTs (a) and (b) COOH functionalized MWCNTs in surfactant.

Figure 4(a) shows the typical SEM image of the MWCNTs before dispersing in water; it showed that MWCNTs were gathered into bundles. After dispersing in water by Tween-80 surfactant and ultrasonic vibration method, the SEM image of MWCNTs showen in Figure 4(b) clearly showed that MWCNTs were not gathered as crowd together.

We measured directly the temperature of the *μ*-processor during the operation of the computer in full load mode (100% usage of *μ*-processor mode). To estimate the role of the liquid, we investigated the temperature of the *μ*-processor when using cooling fan.

**Figure 5:**
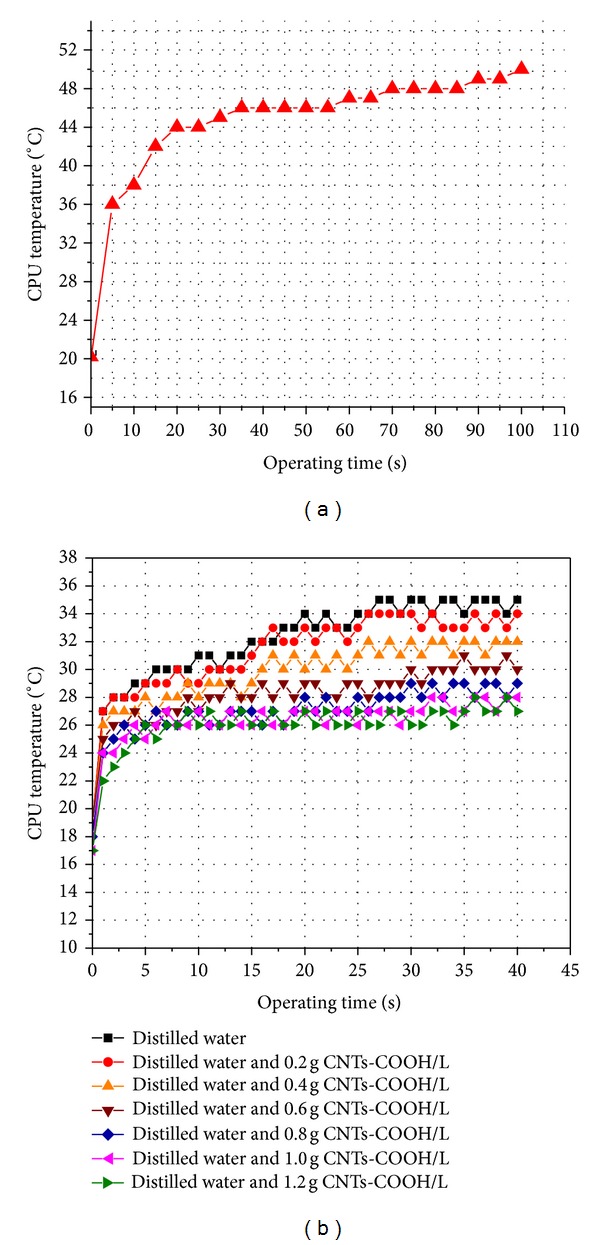
The measured temperature of the *μ*-processor as a function of operation time in the case of using cooling fan (a) and in the case of using CNTs-COOH liquid for thermal dissipation (b).

Figure 5(a) shows result of the *μ*-processor's temperature as a function of working time when using cooling fan. As seen in Figure 5(a), at initial time, the temperature of the *μ*-processor was 20°C, and then the temperature of the *μ*-processor was saturated at approximately 50°C after 100 seconds working time. In order to reduce the saturated temperature and slow down the increasing temperature process of the processor, we used CNTs-COOH liquid in the thermal dissipation process for the *μ*-processor.

Figure 5(b) is the experimental measured result of the *μ*-processor's temperature as a function of working time when using distilled water and CNTs-COOH/distilled water for thermal dissipation. At initial time, the temperature of the *μ*-processor was about 15–18°C. The saturated temperature of the processor reached 35°C, 30°C, and 28°C when using distilled water, 0.6 g CNTs-COOH/litter distilled water and 1 g CNTs-COOH/litter distilled water after 30-minute working time, respectively. These results indicated that in comparison to cooling fan, the saturated temperature of the processor decreased by about 15°C–22°C and increasing temperature time was prolonged from 100 seconds to 30 minutes. By mixing CNTs-COOH (1 g/L) in the distilled water, the saturated temperature of CPU decreased by 7°C, compared to distilled water.  Table 1 summarized the saturated temperature of microprocessor by different thermal dissipation media. These results confirm the thermal dissipation efficiency by adding CNTs, a super high thermal conductivity material, into the thermal conducing materials.

**Figure 6:**
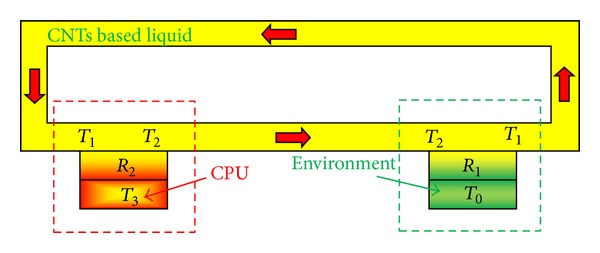
Model of CPU thermal dissipation system using liquid.

To evaluate the thermal dissipation efficiency in the *μ*-processor, we proposed a thermal dissipation system model as shown in Figure 6. In Figure 6,

*T*_0_: temperature of the environment (°C),
*T*_1_: temperature of the liquid flowing to the Cu substrate (°C),
*T*_2_: temperature of the liquid flowing out of the Cu substrate (°C),
*T*_3_: temperature of the *μ*-processor (°C),
*R*_1_: heat resistance between the *μ*-processor and the liquid (K/W),
*R*_2_: heat resistance between the liquid and the environment (K/W).

Heat-flow from the CPU to the liquid and from the coolant to the environment was given by the following expressions, respectively:

(1) $$\begin{array}{c} I_{1}=\frac{T_{3}-\left(\left(T_{1}+T_{2}\right)/2\right)}{R_{2}}\;\;I_{2}=\frac{\left(\left(T_{1}+T_{2}\right)/2\right)-T_{0}}{R_{1}}. \end{array}$$

When liquid flowed through the copper substrate, the heat-flow was given by the following expression:

(2) $$\begin{array}{c} J=\frac{mC\left(T_{2}-T_{1}\right)}{t}=F\cdot D\cdot C\cdot \left(T_{2}-T_{1}\right). \end{array}$$

When the thermal dissipation process reached the saturation state, we have

(3) $$\begin{array}{c} P=I_{1}=J=I_{2}, \end{array}$$

where

*C*: specific heat capacity of the liquid (J/kg · K),
*D*: density of the liquid (kg/m^3^),
*F*: flow-rate of the liquid (m^3^/s),
*P*: heat-generating power of the *μ*-processor (W),
*I*_1_: heat-flow from the CPU to the liquid (W),
*I*_2_: heat-flow from the liquid to the environment (W),
*J*: heat-flow in the liquid (W).

In this work, flow-rate of the liquid in the thermal dissipation system was 2 (cm^3^/s). We also determined the specific heat capacity and density of distilled water that were 4185.5 (J/kg · K) and 999.97 (kg/m^3^), respectively.

When using distilled water, *T*_0_ = 15°C, *T*_1_ = 20°C, *T*_2_ = 27°C, and *T*_3_ = 35°C, heat-generating power of the *μ*-processor was

(4) $$\begin{array}{c} P=V\cdot D\cdot C\cdot \left(T_{2}-T_{1}\right)=58.6\;\text{W}. \end{array}$$

Heat resistance between the *μ*-processor and the liquid and heat resistance between the liquid and the environment were, respectively:

(5) $$\begin{array}{c} R_{1}=\frac{\left(\left(T_{1}+T_{2}\right)/2\right)-T_{0}}{P}=0.145\;\text{K}/\text{W},\\ R_{2}=\frac{T_{3}-\left(\left(T_{1}+T_{2}\right)/2\right)}{P}=0.196\;\text{K}/\text{W}. \end{array}$$

When using 1 g CNTs-COOH/litter distilled water, *T*_0_ = 15°C, *T*_1_ = 18,5°C, *T*_2_ = 25°C, and *T*_3_ = 28°C, heat resistance between the *μ*-processor to the liquid and heat resistance between the liquid to the environment were, respectively:

(6) $$\begin{array}{c} R_{1}=\frac{\left(\left(T_{1}+T_{2}\right)/2\right)-T_{0}}{P}=0.115\;\text{K}/\text{W},\\ R_{2}=\frac{T_{3}-\left(\left(T_{1}+T_{2}\right)/2\right)}{P}=0.107\;\text{K}/\text{W}. \end{array}$$

Specific heat capacity of the CNTs-COOH/distilled water was

(7) $$\begin{array}{c} C_{\text{CNTs}-\text{COOH}/{\text{H}}_{2}\text{O}}=\frac{P}{V\cdot D\cdot \left(T_{2}-T_{1}\right)}=4490\left(\text{J}/\text{kg}\cdot \text{K}\right). \end{array}$$

## 4. Conclusion

We have successfully dispersed MWCNTs into distilled water by using Tween-80 surfactant and ultrasonic vibration method, and the optimal ultrasonic vibration time is 30 minutes. The thermal dissipation efficiency of the PC's *μ*-processor using the cooling fan and liquid cooling was examined and evaluated. Compared to the cooling fan, the saturated temperature of the processor using distilled water decreased by about 15°C. By mixing MWCNTs-COOH (1 g/L) into the distilled water, the saturated temperature of CPU decreased by 7°C compared to only using distilled water. The theoretical results showed that the presence of MWCNTs reduces thermal resistance and increases the thermal conductivity of water from 4185.5 J/kg · K to 4490 J/kg · K. The experimental results have confirmed the advantage of the MWCNT as an excellent additive component in liquid for the thermal dissipation media of computer processors or high power electronic devices.

## Figures and Tables

**Table 1:** The saturated temperature of microprocessor by different thermal dissipation media.

Thermal dissipation method	Saturated temperature of the *μ*-processor
Cooling fan method	50°C
Distilled water	35°C
CNT based liquid (0.2 g MWCNTs-COOH/litre)	34°C
CNT based liquid (0.4 g MWCNTs-COOH/litre)	32°C
CNT based liquid (0.6 g MWCNTs-COOH/litre)	31°C
CNT based liquid (0.8 g MWCNTs-COOH/litre)	29°C
CNT based liquid (1.0 g MWCNTs-COOH/litre)	28°C
CNT based liquid (1.2 g MWCNTs-COOH/litre)	28°C
